# Insulin-positive ductal cells do not migrate into preexisting islets during pregnancy

**DOI:** 10.1038/s12276-021-00593-z

**Published:** 2021-04-05

**Authors:** Qun Liu, Yinan Jiang, Lingyan Zhu, Jieqi Qian, Chaoban Wang, Tianlun Yang, Krishna Prasadan, George K. Gittes, Xiangwei Xiao

**Affiliations:** 1 Department of Endocrinology, The First Affiliated Hospital of NanChang University, Nanchang, 330006 China; 2 Department of Surgery, Children’s Hospital of Pittsburgh, University of Pittsburgh School of Medicine, Pittsburgh, PA 15224 USA; 3 Department of Pediatric Endocrinology, The Second Affiliated Hospital and Yuying Children’s Hospital of Wenzhou Medical University, Wenzhou, 325000 China; 4 Department of Cardiology, Xiangya Hospital, Central South University, Changsha, 410078 China

**Keywords:** Transdifferentiation, Gestational diabetes

## Abstract

The adult pancreatic ductal system was suggested to harbor facultative beta-cell progenitors similar to the embryonic pancreas, and the appearance of insulin-positive duct cells has been used as evidence for natural duct-to-beta-cell reprogramming. Nevertheless, the phenotype and fate of these insulin-positive cells in ducts have not been determined. Here, we used a cell-tagging dye, CFDA-SE, to permanently label pancreatic duct cells through an intraductal infusion technique. Representing a time when significant increases in beta-cell mass occur, pregnancy was later induced in these CFDA-SE-treated mice to assess the phenotype and fate of the insulin-positive cells in ducts. We found that a small portion of CFDA-SE-labeled duct cells became insulin-positive, but they were not fully functional beta-cells based on the in vitro glucose response and the expression levels of key beta-cell genes. Moreover, these insulin-positive cells in ducts expressed significantly lower levels of genes associated with extracellular matrix degradation and cell migration, which may thus prevent their budding and migration into preexisting islets. A similar conclusion was reached through analysis of the Gene Expression Omnibus database for both mice and humans. Together, our data suggest that the contribution of duct cells to normal beta-cells in adult islets is minimal at best.

## Diabetes: Tracing cells that might transform to produce insulin

The tantalizing possibility that the adult pancreas harbors cells that could transform into insulin-producing cells called beta cells appears unlikely. Past research indicated that cells lining the ducts of the pancreas had the potential to differentiate into beta cells, which produce insulin to control blood sugar. Using a mouse model, Xiangwei Xiao at the University of Pittsburgh School of Medicine, USA, and Lingyan Zhu at the First Affiliated Hospital of NanChang University, China, tagged pancreatic duct cells with a special dye, then traced them to see if they became functioning beta cells. Although the cells did produce trace amounts of insulin, they did not produce insulin in response to glucose, nor express all the genes expressed by a mature beta cell. These results enhance our understanding of beta cell development and insulin production.

## Introduction

The short supply of donor pancreases prevents extensive clinical application of islet transplantation as a cure for diabetes^1–3^, which heightens the need for alternative sources of insulin (INS)-producing beta-cells. Beta-cell expansion in adults is mainly attributable to beta-cell replication. However, since mature beta-cells have a very slow proliferation rate^4^, which further declines with age^5–8^, great efforts have been made to find non-beta-cell sources for generating functional beta-cells^9^.

Adult pancreatic duct cells have been extensively studied for their potential to generate functional beta-cells, since embryonic pancreatic ductal structures clearly harbor endocrine precursors^10–12^. However, accumulating evidence from lineage tracing casts significant doubt on this possibility^13^. On the other hand, the tamoxifen-induced CreERT/loxp-based lineage tracing of duct cells has been shown to be very capricious and potentially unreliable due to highly variable expression of the promoter used to drive Cre recombinase^14–17^. This variability is particularly problematic when the conclusions are that a very small number of cells change their phenotype^14–17^. Therefore, numerous conflicting results have been generated using Cre-mediated lineage tracing strategies in ducts^13^. Nevertheless, it is a core belief that the adult pancreatic ductal system also harbors facultative beta-cell progenitors similar to the embryonic pancreas, and the documentation of INS+ ductal cells was repeatedly used to support possible duct-to-beta-cell reprogramming^9^. However, the phenotype and fate of these INS+ cells in ducts have not been determined.

Here, we used a cell-tagging dye, carboxyfluorescein diacetate succinimidyl ester (CFDA-SE), to permanently label pancreatic duct cells through an intraductal infusion technique. We have previously shown that this method is efficient, specific, and durable^18,19^. Pregnancy is a well-accepted model for physiologic beta-cell growth in adult mice in which beta-cell mass nearly doubles at its peak^20^. Thus, pregnancy was later induced in these CFDA-SE-treated mice to assess the phenotype and fate of the insulin-positive cells in ducts.

We found that a small portion of CFDA-SE-labeled duct cells became insulin-positive, but they were not fully functional beta-cells based on the in vitro glucose response and the expression levels of key beta-cell genes. Moreover, these insulin-positive cells in ducts, as well as preexisting beta-cells in islets in adult mice, expressed significantly lower levels of genes associated with extracellular matrix degradation and cell migration, which may explain their failure to exit from the ducts and migrate to preexisting islets. A similar conclusion was obtained through analysis of the Gene Expression Omnibus (GEO) database from both mice and humans.

## Materials and methods

### Mouse manipulation

All mouse experiments were approved by the Animal Research and Care Committee at the Children’s Hospital of Pittsburgh and the University of Pittsburgh IACUC. C57/BL6, INS1-Cre knock-in^21^, and Rosa26CAGTomato (Tomato)^22^ mice were all purchased from the Jackson Lab (Bar Harbor, MA, USA). INS1-Cre knock-in mice were bred with Tomato mice to generate Ins1^cre^;R26^tomato^ mice for visualization or purification of beta-cells based on red fluorescence. Due to the need for pregnancy, only female mice at 10–12 weeks of age were used for experiments. Pancreatic intraductal infusion of CFDA-SE was performed as described^19^. Three weeks after CFDA-SE infusion, the incision on these mice healed. Then, these mice were time-mated with male C57BL/6 mice. Pregnant mice were analyzed at gestational day 16 (G16).

### Pancreatic digestion and FACS

Pancreatic duct perfusion and subsequent digestion of the pancreas were performed as described previously^23^. Islet isolation was performed as described previously^24^. Briefly, the mice were sacrificed with CO_2_, and then the upper abdomen was exposed for pancreatic duct perfusion and subsequent digestion of the pancreas with 0.2 mg/ml collagenase (Sigma-Aldrich, Pittsburgh, PA, USA). The islets were hand-selected, followed by further digestion with 10 μg/ml trypsin and 10 μg/ml DNase (Sigma-Aldrich) for ~20 min to obtain a single islet cell population, which was used to purify beta-cells using flow cytometry based on direct fluorescence of Tomato (Ins1^cre^;R26^tomato^ mice). The nonislet fraction was also digested with 10 μg/ml trypsin and 10 μg/ml DNase for approximately 20 minutes to obtain a single nonislet cell population, which was either incubated with a duct-binding lectin, fluorescein Dolichos biflorus agglutinin (DBA, Vector Lab, Burlingame, CA), for 20 min for purification of green DBA^+^ duct cells by FACS or directly analyzed for green CFDA-SE fluorescence in CFDA-SE-infused mice. For islet perfusion for the glucose tolerance test, purified beta-cells were reaggregated for 2 h and cultured overnight before analysis, as described previously^25–27^.

### RNA isolation and RT-qPCR

Total RNA was extracted using an RNeasy mini kit (Qiagen, Valencia, CA, USA) and then quantified with a Nanodrop 1000 (Thermo Fisher Scientific, Inc., Waltham, MA, USA), followed by cDNA synthesis (Qiagen) and RT-qPCR. The primers were all purchased from Qiagen. Relative values of mRNA levels were obtained by sequential normalization against the housekeeping gene cyclophilin A and the experimental control.

### Immunohistochemistry

All pancreas samples were fixed in zinc (BD) for 4 h followed by an additional 2 h of fixation in 4% formaldehyde and then were cryoprotected in 30% sucrose overnight before freezing. Whole-mount staining was done as described^28^. Tomato and CFDA-SE were detected by direct fluorescence. DAB (3,3′-diaminobenzidine) staining was performed with the ABC method. The primary antibody for immunostaining was guinea pig polyclonal insulin (Dako, Carpinteria, CA, USA). The secondary antibodies for indirect fluorescent staining were Cy2-, Cy3-, or Cy5-conjugated donkey streptavidin- and guinea pig-specific (Dako). Nuclear staining was performed with DAPI (4′,6-diamidino-2-phenylindole; Dako).

### Quantification

The quantification of FACS was performed objectively by machine using consistent settings and gating. The quantification on slides was performed according to at least 5 sections that were 100 µm apart to avoid the possibility of analyzing one islet twice. Since the absolute number of positive cells was small, at least 10,000 cells were counted for each experimental condition. If the percentage of positive cells was very low, counting continued beyond 10,000 cells to ensure that at least 30 positive cells were counted in each repeat.

### Data analysis

For in vivo experiments, 5–8 mice were used for each group. All data were statistically analyzed by one-way ANOVA with a Bonferroni correction, followed by Fisher’s exact test. All error bars represent S.D. (standard deviation). Significance was presented as * when *p* < 0.05. No significance was presented as NS. *P* values and *n* values are indicated in the figure legends. For bioinformatics analysis, transcriptome RNA-sequencing (RNA-seq) data of mouse and human beta-cells from different ages were downloaded from the GEO data portal (https://www.ncbi.nlm.nih.gov/geo/). RNA-seq data of 4–5 specimens in each group (GSE54374 for mice and GSE67543 for humans) were used for analysis by the R software Linear Models for Microarray and RNA-Seq Data (Limma) package. Pairwise differential expression was quantified using Cuffdiff. Cufflinks was used to determine FPKM levels for each gene from the STAR alignment and was used as input for Cuffdiff. Next, we performed differential gene analysis of all transcriptional data, setting a log2 |fold change|>1 and a false discovery rate (FDR) < 0.05 as the cutoff values.

## Results

### Increased INS+ cells in pancreatic ducts during pregnancy

**Fig. 1:**
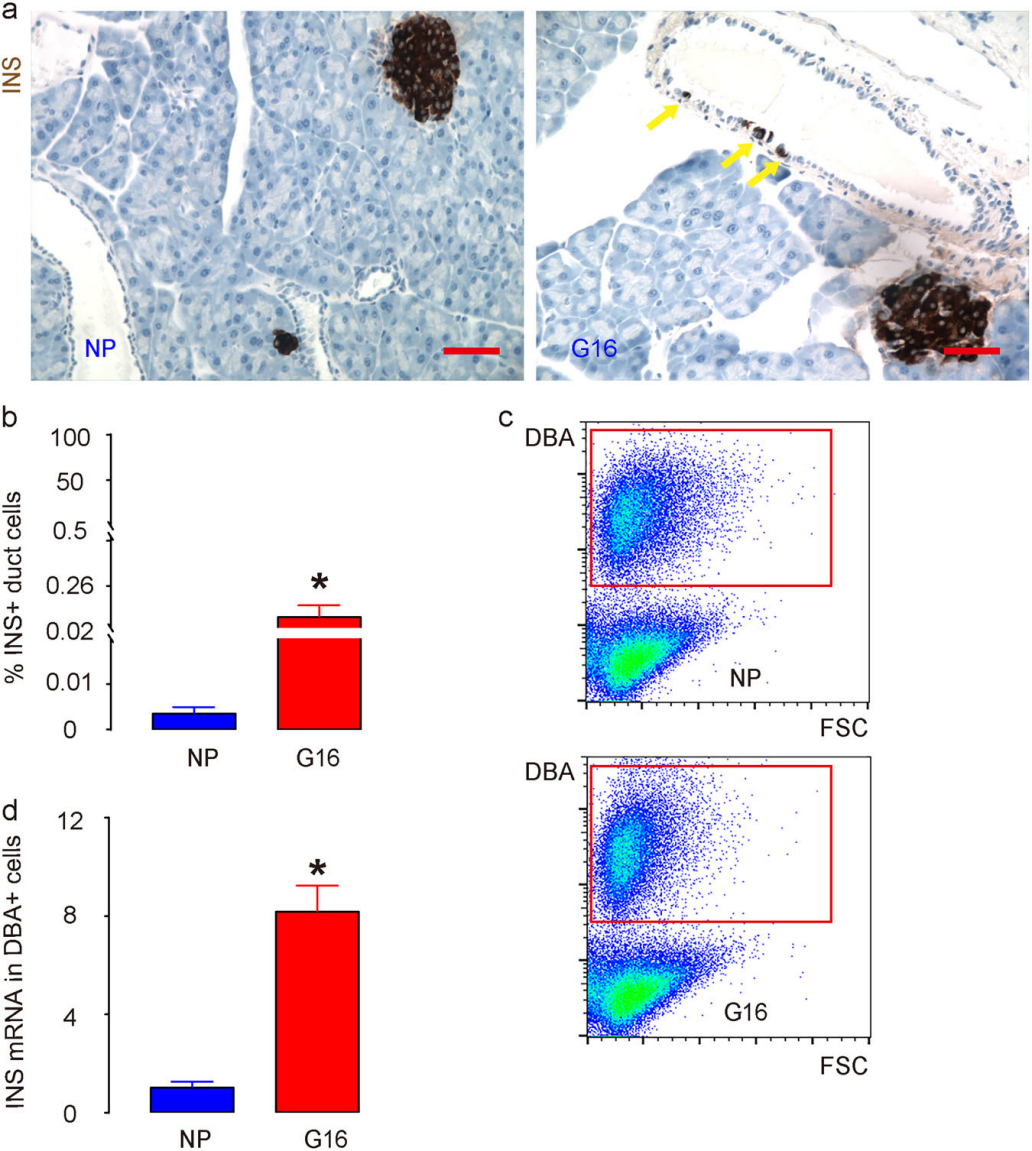
Increases in INS+ cells in pancreatic ducts during pregnancy. The existence and number of INS+ cells from pancreatic ducts were assessed at gestational day 16 (G16) compared to nonpregnant (NP) mice. **a**, **b** Quantification of INS+ cells in pancreatic ducts by immunohistochemistry, shown by representative images (**a**) and by quantification (**b**). **c** Representative flow charts for isolating DBA+ duct cells from the pancreas of NP and G16 mice. **d** RT-qPCR for INS in DBA+ duct cells from NP and G16 mouse pancreases. Yellow arrows point to INS+ cells in the pancreatic ducts. *P < 0.05; *N* = 5. Scale bars are 50 µm.

During pregnancy, pancreatic beta-cells undergo a significant expansion in response to metabolic needs, and this increase in beta-cells predominantly occurs late in gestation, around day 16 (G16)^23^. Hence, we selected G16 to analyze INS+ cells derived from pancreatic ducts. First, we examined the existence and number of INS+ cells in pancreatic ducts at G16 compared to nonpregnant (NP) mice. We detected a significantly higher number of INS+ cells in pancreatic ducts, as shown by representative images (Fig. 1a, Supplementary Figure) and quantification (Fig. 1b). Previous studies have also shown that very few INS+ cells are detected in the normal adult pancreatic duct, and these rare cells have been suggested to be vestiges of the embryonic pancreas, where some beta-cells fail to bud from the duct trunk, possibly due to inadequate expression of budding-dependent gene clusters at the single-cell level. However, the significant increase in the number of INS+ cells in pancreatic ducts that we found during pregnancy suggests that the INS promoter of some duct cells may be specifically activated in adulthood under certain circumstances, such as hormonal induction. We isolated DBA+ duct cells from the pancreas of NP and G16 mice (Fig. 1c) and found that the INS mRNA levels in duct cells were significantly increased in the pancreas of G16 mice compared to the pancreas of NP mice (Fig. 1d). Together, these data suggest increases in INS+ cells in pancreatic ducts during pregnancy.

### Labeling of pancreatic duct cells with CFDA-SE

**Fig. 2:**
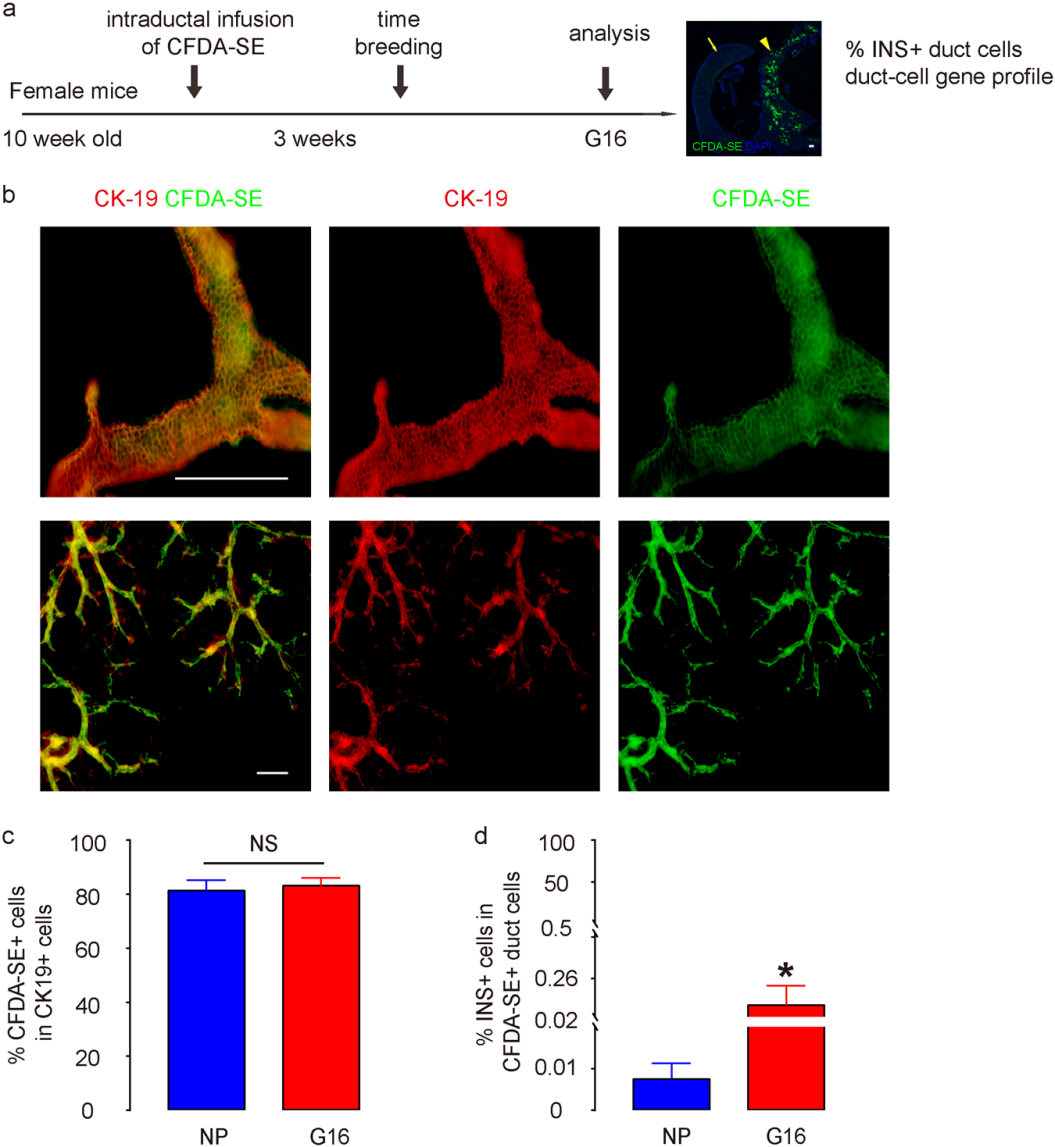
Labeling of pancreatic duct cells with CFDA-SE. **a** Schematic of the labeling and tracing of CFDA-SE-labeled duct cells. We applied an intraductal infusion technique to label pancreatic duct cells with CFDA-SE in 10-week-old female C57BL/6 mice. After 3 weeks, when the incision on these mice was healed, these mice were time-mated with male C57BL/6 mice. The pregnant mice were sacrificed at G16 to analyze the percentage of INS+ duct cells and the duct cell gene profile. **b** Representative whole-mount imaging of the mouse pancreas 1 week after CFDA-SE infusion, showing that large ducts (upper panel) and branch ducts (lower panel) were both well labeled. CK19 is a pan-duct marker. **c** Quantification of the percentage of CK19+ cells that were labeled with CFDA-SE. **d** After staining with INS, the percentage of INS+ cells in the total CFDA-SE+ cells was quantified. The arrow points to the spleen, and the arrowhead points to the pancreas. *P < 0.05; NS: no significance. *N* = 5. Scale bars are 100 µm.

Next, we applied an intraductal infusion technique to efficiently, specifically, and durably label pancreatic duct cells with CFDA-SE, a dye that passively enters the cells and is then retained within the cells^18,19,29^. CFDA-SE was infused into the pancreas from the pancreatic duct to specifically label duct cells in 10-week-old female C57BL/6 mice, as described^18,19,29^. After 3 weeks, when the incision on these mice was healed, these mice were time-mated with male C57BL/6 mice. The pregnant mice were sacrificed at G16^20,23^ to analyze the percentage of INS+ duct cells and the duct cell gene profile (Fig. 2a). From 1 week after infusion, the CK19+ pancreatic ducts were readily visualized by CFDA-SE (in green) by whole-mount imaging (Fig. 2b). We found that the green CFDA-SE signal colocalized faithfully with the red CK19 (duct marker) signal by immunostaining (Fig. 2b). More than 80% of CK19+ duct cells were successfully labeled by CFDA-SE (Fig. 2c), and CK19-/CFDA-SE+ cells were nearly nonexistent (less than 2 in 10^4^ cells). We found that the percentage of INS+ cells out of the total CFDA-SE+ cell population was significantly higher in the pancreas of G16 mice than in the pancreas of NP mice (Fig. 2d), which was consistent with our quantification of the percentage of INS+ duct cells based on DBA/INS staining (Fig. 1a, b).

### INS+ cells in pancreatic ducts during pregnancy do not migrate into preexisting islets

**Fig. 3:**
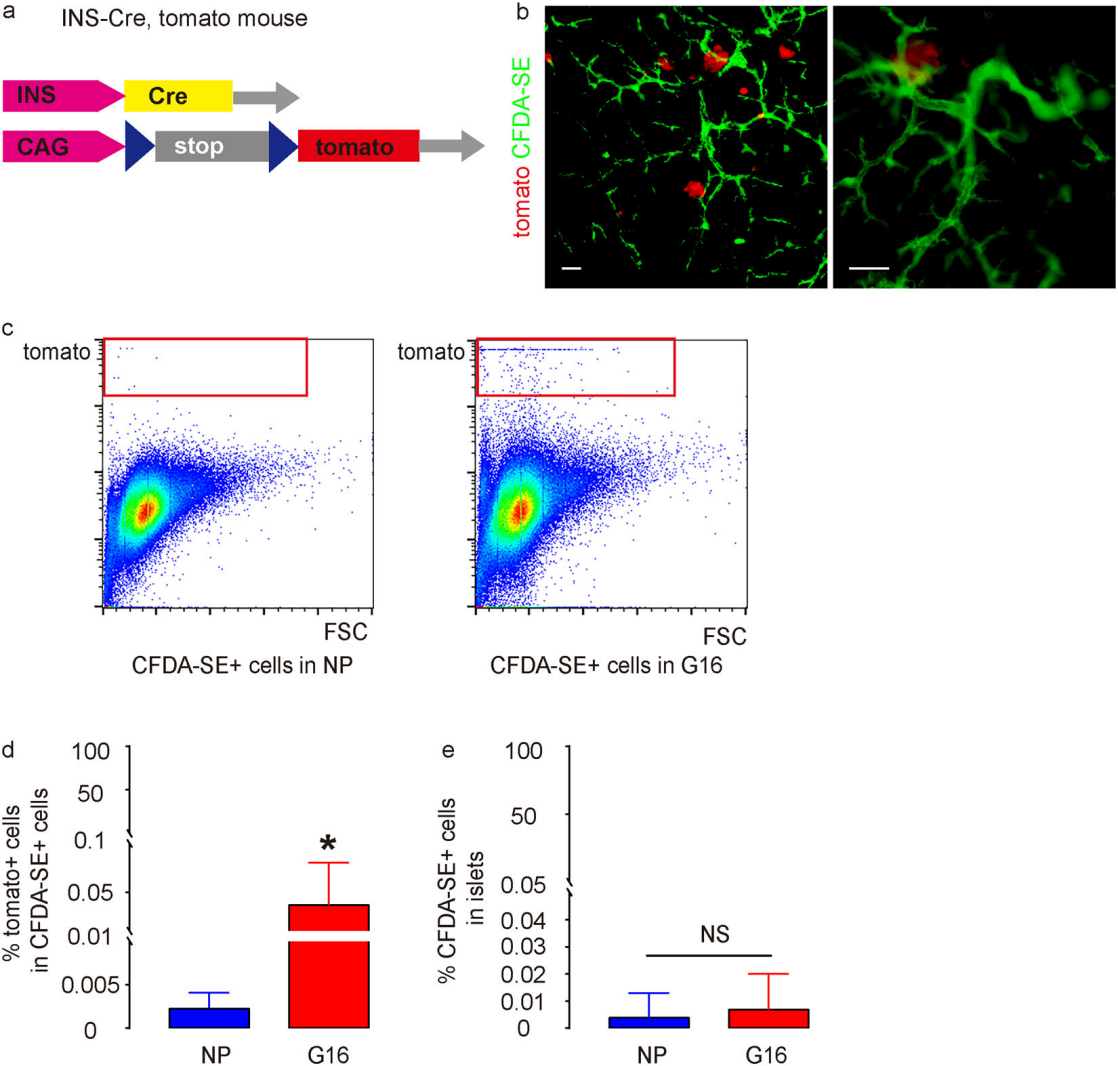
INS+ cells in pancreatic ducts during pregnancy do not migrate into preexisting islets. **a** Schematic of the Ins1^cre^;R26^tomato^ mouse model. **b** Representative whole-mount imaging of the pancreas of Ins1^cre^;R26^tomato^ mice 1 week after CFDA-SE infusion. **c** Representative flow charts for isolating Tomato+ cells from the CFDA-SE+ ductal fraction from the NP and G16 Ins1^cre^;R26^tomato^ mouse pancreases. **d** Quantification of the percentage of Tomato+ cells in total CFDA-SE-labeled ductal cells by FACS. **e** Quantification of the percentage of CFDA-SE+ cells in islets by section. *P < 0.05; NS: no significance. *N* = 5. Scale bars are 100 µm.

To assess the phenotype and fate of the INS+ cells in ducts, we used an INS-Cre Tomato red reporter mouse model (Ins1^cre^;R26^tomato^) to perform the same duct cell tracking experiment as schematized in Fig. 2a. INS1-Cre is a knock-in mouse strain, and it was bred with a Tomato reporter strain to allow visualization or purification of beta-cells based on red fluorescence (Fig. 3a). CFDA-SE intraductal infusion generated similar ductal labeling in these mice as in Fig. 2, but here they bear red islets due to the Ins1^cre^;R26^tomato^, shown by whole-mount imaging (Fig. 3b). Tomato+ cells were purified from CFDA-SE+ pancreatic cells from NP and G16 mice, and thus, these cells represented INS+ cells either from ducts or from duct cells that had moved out of the ductal wall (Fig. 3c). We detected significantly more Tomato+ cells in the CFDA-SE+ fraction from pregnant mice than from controls (Fig. 3d), again confirming our data in Figs. 1a, b and 2d. Interestingly, despite this significant increase in Tomato+ cells in the CFDA-SE+ fraction, we detected few if any CFDA-SE+ cells in the mouse islets, either in NP or G16 mice (Fig. 3e). This number did not increase at 4 weeks after delivery of the pregnant mice, suggesting that the INS+/CFDA-SE+ did not simply need more time to get to the islets. Thus, INS+ cells in pancreatic ducts during pregnancy do not appear to migrate into preexisting islets to a detectable level.

### INS+ cells in ducts are not fully functional beta-cells

**Fig. 4:**
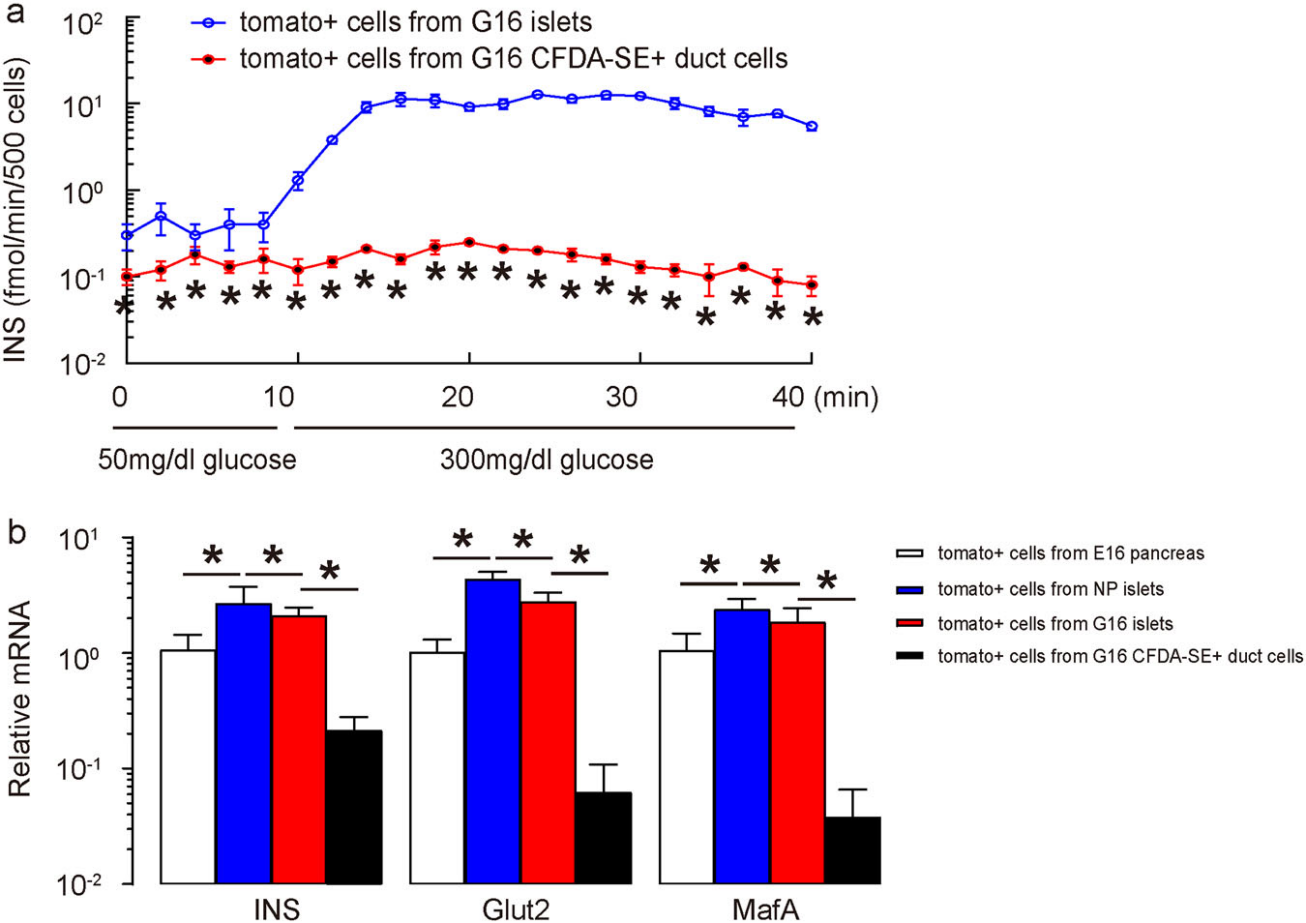
INS+ cells in ducts are not fully functional beta-cells. **a** The small proportion of Tomato+/CFDA-SE-labeled duct cells purified by FACS was then reaggregated overnight and subjected to a glucose tolerance test. Compared to Tomato+ cells purified from G16 islets, Tomato+ cells purified from CFDA-SE+ duct cells from G16 mouse pancreas exhibited much lower basal INS release and almost no glucose-stimulated INS release. **b** RT-qPCR for INS, Glut2, and MafA in FACS-purified beta-cells. *P < 0.05; *N* = 5.

Next, we assessed the phenotype of these INS+ cells in ducts. A small portion of Tomato+/CFDA-SE+ duct cells was then purified by FACS, reaggregated overnight, and then subjected to a glucose-stimulated insulin secretion test. We found that compared to Tomato+ cells purified from G16 islets, Tomato+ cells purified from CFDA-SE+ duct cells from G16 mouse pancreas exhibited much lower basal INS release and almost no glucose-stimulated INS release (Fig. 4a). Moreover, these Tomato+ cells purified from CFDA-SE+ duct cells from G16 mouse pancreas expressed much lower levels of INS, Glut2, and MafA, genes that are critical for mature beta-cell function (Fig. 4b). These data thus suggest that these INS+ cells in ducts were not fully functional beta-cells and perhaps were just occasional duct cells that had turned on the insulin promoter without undergoing a complete beta-cell differentiation process.

### INS+cells in ducts express low levels of genes associated with extracellular matrix degradation and cell migration

**Fig. 5:**
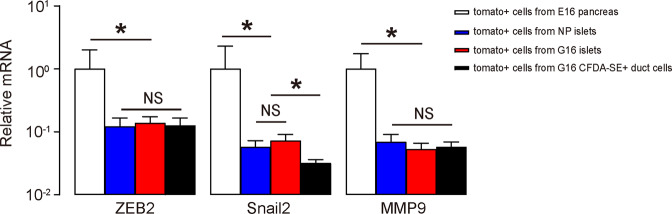
INS+ cells in ducts express low levels of genes associated with extracellular matrix degradation and cell migration. RT-qPCR for ZEB2, Snail2, and MMP9 in FACS-purified beta-cells. *P < 0.05; NS: no significance. *N* = 5.

Since we detected increases in INS+ cells in ducts at G16 but we did not detect increases in INS+/CFDA-SE-labeled cells later in the mouse islets, it appeared that these cells did not bud from the ducts and subsequently migrate into islets, as they may have in the embryonic pancreas. We thus hypothesized that these cells may express lower levels of certain genes necessary for cell migration or degradation of extracellular matrix (ECM). From a selected group of such genes, we found that ZEB2, Snail2 and MMP9 were expressed at significantly lower levels in Tomato+/CFDA-SE+ duct cells from G16 mouse pancreas compared to Tomato+ true beta-cells purified from E16 mouse islets or whole pancreas (Fig. 5). The low expression of such migration-related factors may account for the failure of INS+ duct cells to bud off of the ducts to join the islets. Interestingly, Tomato+ cells from adult mouse islets from either NP or G16 mice also expressed lower levels of these genes than Tomato+ cells purified from gestational day 16 embryonic (E16) mouse pancreas (Fig. 5). These data suggest that adult beta-cells may have less migratory potential than embryonic beta-cells.

### Bioinformatic analyses confirm that both mouse and human beta-cells in the adult pancreas express low levels of genes associated with ECM degradation and cell migration compared to beta-cells in the embryonic pancreas

**Fig. 6:**
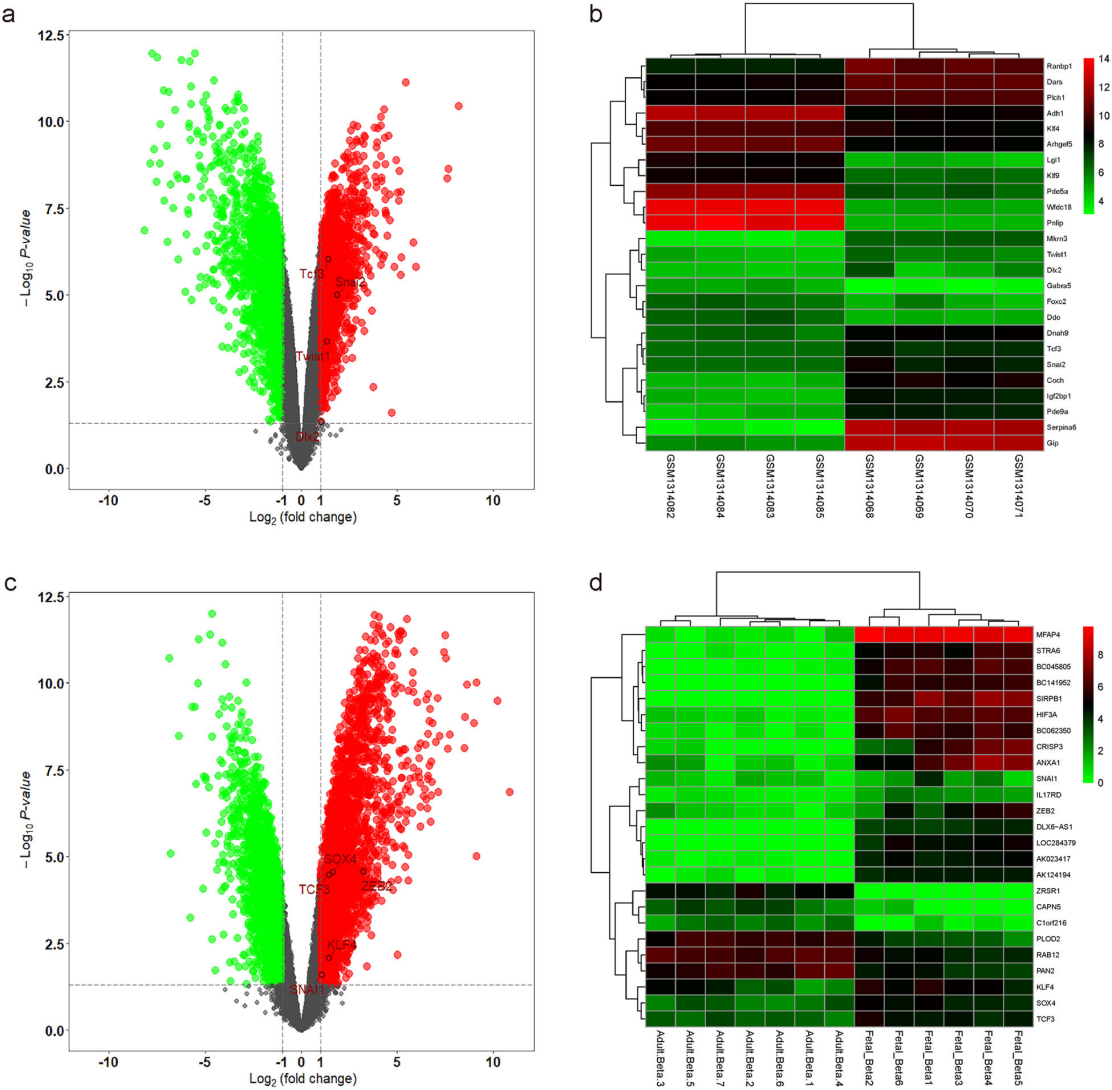
Bioinformatic analysis shows that both mouse and human beta-cells in the adult pancreas express low levels of genes associated with ECM degradation and cell migration compared to beta-cells in the embryonic pancreas. **a**, **b** Volcano image (**a**) and heat map (**b**) for GEO database GSE54374 on Platform GPL1261 to compare gene profiling of INS+ cells from embryonic mice to adult mice. Snail2, TCF3, Twist1, and Dlx2 were expressed at lower levels in adult mouse beta-cells. **c**, **d** Volcano image (**c**) and heat map (**d**) for GEO database GSE67543 on Platform GPL11154 to compare gene profiling of INS+ cells from embryonic humans to adult humans. ZEB2, SOX4, TCF3, KLF4, and Snail 1 were expressed at lower levels in adult human beta-cells. Data were analyzed by R language.

Finally, we performed GEO database mining and bioinformatics analyses to validate our findings. First, we reanalyzed the data from GSE54374 on Platform GPL1261^30^, which allowed us to compare the gene profiling of beta-cells from embryonic mice to that of adult mice. The Limma R package identified some genes involved in ECM degradation and cell migration, which were all expressed at significantly lower levels in adult beta-cells than in embryonic beta-cells, including Snail2, TCF3, Twist1, and Dlx2, as shown in a volcano map (Fig. 6a) and in a heat map (Fig. 6b). Interestingly, changes in Snail2 expression were similarly shown in our own analysis (Fig. 5). Next, we reanalyzed the data from GSE67543 on Platform GPL11154^31^, which allowed us to compare the gene profiling of beta-cells from embryonic humans to adult humans. The Limma R package identified some genes involved in ECM degradation and cell migration, which were all expressed at significantly lower levels in adult human beta-cells than in embryonic human beta-cells, including ZEB2, SOX4, TCF3, KLF4, and Snail 1, as shown in a volcano map (Fig. 6c) and in a heat map (Fig. 6d). Interestingly, ZEB2 was similarly detected in our own study (Fig. 5). Together, these bioinformatic analyses from previous studies independent of ours suggest that the decreased migrating potential of adult beta-cells from both mice and humans may be due to their expression of low levels of genes associated with ECM degradation and cell migration compared to INS+ cells in the embryonic pancreas.

## Discussion

Past genetic lineage-tracing experiments have demonstrated that the ductal trunk in the embryonic pancreas harbors multipotent progenitors for exocrine, endocrine, and duct cells^32^. Developing embryonic beta-cells bud and detach from these trunks/ducts and subsequently migrate to the islet niche to form islets. It has been speculated that this formation of new beta-cells from ductal progenitor cells may be recapitulated in adults^9^. However, accumulating evidence has refuted this possibility. Dor and Melton^33^, Teta and Kushner^34^, and our group^23^ independently showed a minimal contribution of beta-cell neogenesis in the adult pancreas, each with an innovative technology. It thus appears that direct gene manipulation is required for duct-to-beta-cell reprogramming. For example, Grompe and colleagues used a cocktail of Pdx1, MafA, and Ngn3 to generate beta-like cells from adult duct cells^35^. Kulkarni and colleagues recently showed that knockout of a liver-specific insulin receptor led to reprogramming of human duct cells into beta-like cells^36^. In another report, Kim and colleagues have nicely shown that gene therapy to reprogram adult pancreatic duct cells into beta-like cells requires not only Pdx1, MafA, and Ngn3, three of which are capable of reprogramming adult acinar cells into beta-like cells^37^ and two of which are capable of reprogramming adult alpha-cells into beta-cells^29,38^, but also an additional transcription factor, Pax6, to fulfill reprogramming^39^. Adult duct cells undergo significant epigenetic modification to stabilize their phenotype (38). Unlike islet cells^40,41^, duct cells are continuously exposed to digestive fluids, which may require a more strictly organized epigenetic signature to render them more resistant to phenotypic change, perhaps also offering beneficial resistance to carcinogenesis.

A second concept supporting beta-cells arising from duct cells (the first being recapitulation of embryonic events) came from lineage tracing studies^13^. However, previous analyses of duct cell lineages with tamoxifen-sensitive Cre-labeling systems have led to great controversy^13,42–46^, which resulted from inaccurate labeling of cells, unstable expression of the gene promoter (typically a transcription factor gene) after tamoxifen-induced Cre activation, prelabeling (“leaky labeling”) of beta-cells, or even cell-type-specific apoptosis^14–16,33,47^. Here, we chemically labeled duct cells by intraductal infusion, which bypassed these issues. Once lipid-soluble CFDA-SE enters the cytoplasm of duct cells, intracellular esterases remove the acetate groups of CFDA-SE and convert it into a non-membrane-permeable fluorescent ester (CFSE) that is retained within the cells^48^. This technique has been widely used as a long-term tracer of cells and as a method of studying cell proliferation^49^. The fluorescent signals will dilute to 50% when a cell splits. In vivo, when used for labeling pancreatic duct cells, which are not highly proliferative, labeling can be maintained for a very long period, which is more than adequate for the purpose of the current study. The findings reported here in pregnancy are consistent with our previous study in a partial ductal ligation model^18^, showing little contribution of duct cells to beta-cells in adults.

A third concept supporting naturally occurring duct-to-beta-cell reprogramming is the presence of INS+ cells in adult pancreatic ducts, especially in diabetes and other conditions that favor beta-cell growth^9^. However, the phenotype and fate of these INS+ cells in ducts have not been addressed in previous studies. Here, we showed that these INS+ cells in ducts were not fully functional, with low-level expression of beta-cell markers, such as INS, Glut2, and MafA. Glut2 and MafA are also critical for proper beta-cell function and phenotype maintenance^50^. Thus, the poor functionality of these INS+ cells in ducts may result from their failure to fully develop into mature beta-cells; they may simply be duct cells that turn on the INS gene but do not undergo beta-cell maturation. Furthermore, by analyzing our data and examining public databases, we found that not only INS+ cells in ducts but also adult beta-cells in islets expressed low levels of genes associated with cell migration and ECM degradation. In the embryonic pancreas, beta-cells budding from the duct trunk presumably need to express these genes to allow them to migrate into an islet niche to form and expand the islets. In adults, this migrating potential of beta-cells decreases, perhaps due not only to the downregulation of these migrating genes but also to the strengthened ECM with laminin and collagens surrounding mature islets^51^. Indeed, the epithelial and mesenchymal properties of beta-cells are critical for their phenotype maintenance, migrating potential, and modulation of functionality, as we reported previously^50^. Thus, without robust data and strategies for real-time tracing of a “reprogrammed” beta-cell outside of an islet, it is only speculation that such cells can degrade mature ECM and migrate through the pancreatic interstitium to join a preexisting islet.

Together, our data suggest that, under normal physiological conditions, including pregnancy, despite the need to expand the beta-cell mass, the duct cells, even the INS+ duct cells, make little or no contribution to the functional beta-cell population. Additionally, adult beta-cells may be unable to migrate due to low expression levels of necessary genes, as well as due to ECM changes that occur during the maturation of the islet microenvironment.

## Supplementary information

Supplementary Figure
