# Supplementary material for: Insulin-positive ductal cells do not migrate into preexisting islets during pregnancy
Source: Exp Mol Med. 2021 Apr 5;53(4):605–14. doi: 10.1038/s12276-021-00593-z (PMC8102600; doi:10.1038/s12276-021-00593-z)
Supplement: Supplementary file 1 — Supplementary Figure [file 12276_2021_593_MOESM1_ESM.pdf]

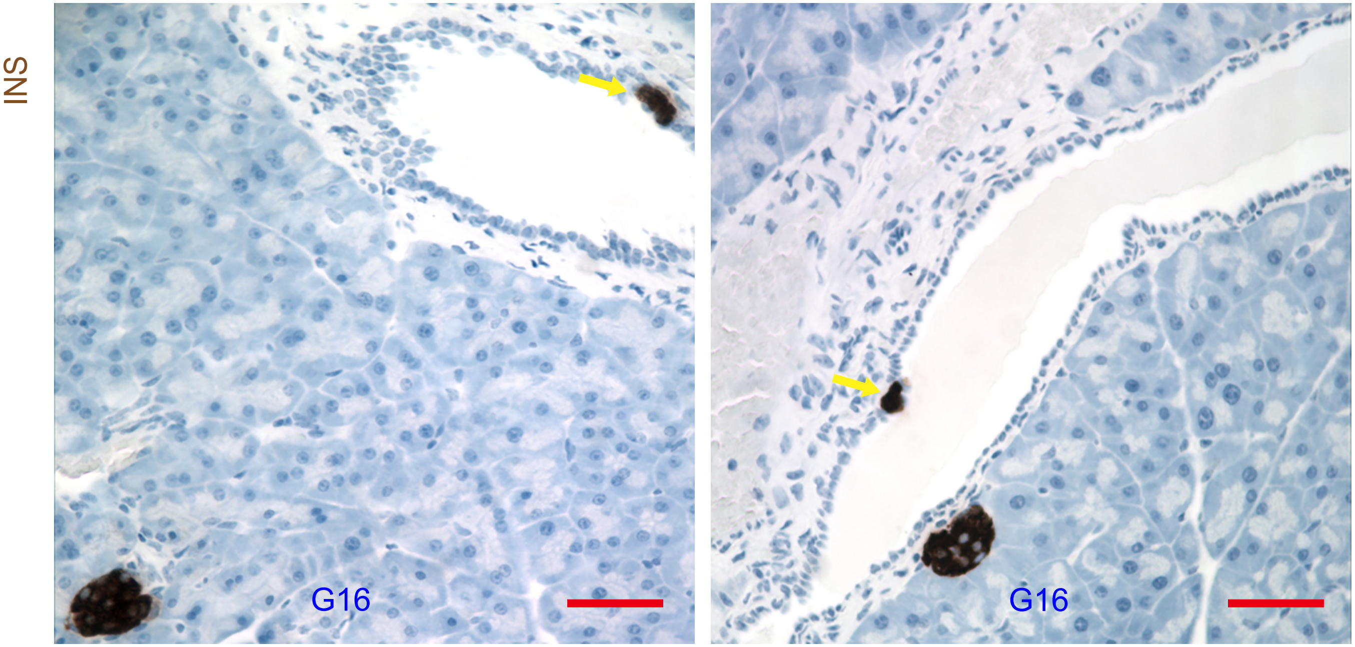

supplementary figure: Increases in INS+ cells in pancreatic ducts during pregnancy
The existence and number of INS+ cells from pancreatic ducts was assessed at gestational day 16 (G16) compared to non-pregnant (NP) mice. Additional representative images at G16 were shown. Yellow arrows pointed to INS+ cells on pancreatic ducts. Scale bars are 50 μm.
